# Developing a framework for culture-based interventions for well-being in Cluj-Napoca, Romania

**DOI:** 10.1186/s40410-022-00182-1

**Published:** 2022-12-06

**Authors:** Claudia-Maria Cacovean, Marina-Denisa Dascăl, Maria-Rarița Zbranca

**Affiliations:** 1 Cluj Cultural Centre, Cluj-Napoca, Romania; 2 Department of Public Health, Faculty of Political, Administrative and Communication Sciences, Babeș-Bolyai University, Cluj-Napoca, Romania

**Keywords:** Well-being, Burnout, Cultural intervention Arts Culture, Urban development, Public policy, Scaling up

## Abstract

The article discusses the context and conditions for implementing culture-based interventions for health and well-being in an urban setting. Specifically, the article presents the case study of a cultural intervention aimed at reducing burnout-related symptoms in adults, describing the context of the intervention, the project design, implementation, and the impact assessment. Following the assessment of the implementation, the next steps in order to scale up the piloted intervention for other people confronting burnout have been identified. Further on, the article analyses the factors that future policies and programmes should take into account for enabling local communities to draw most benefits from the contribution of arts and culture for health and well-being, by proposing possible avenues for scaling up the pilot initiative.

## Introduction

The article presents the case study of a cultural intervention aimed at reducing burnout-related symptoms in adults, carried out in Cluj-Napoca, Romania. It analyses the factors that future policies and programmes should take into account for enabling local communities to draw most benefits from arts for health and well-being, and proposes directions for horizontal and vertical scaling up of the pilot initiative.

In recent years Cluj-Napoca is going through an accelerated expansion and development, way beyond its capacity to manage growth (World Bank 2021). Cluj-Napoca is one of the largest secondary cities in Romania and one of Europe’s youngest cities. Cluj Metropolitan Area has had the fastest economic growth in the EU in the last 20 years according to the World Bank (2021), based on data provided by Eurostat. Besides its positive effects, this accelerated growth comes also with negative externalities such as chaotic urban development, pollution, traffic congestion and higher cost of living (World Bank 2021). The health and well-being challenges that arise require new, innovative and accessible responses. Addressing these issues is significant for the quality of life at both individual and community levels. The city needs to find structural solutions to effectively deal with such challenges in order to ensure that its development is human-centred and sustainable (Zbranca 2018).

Considering the art for health dimension, there are a limited number of initiatives in the city that address health or well-being through art/culture. All relevant activities are currently being carried out at the initiative of various private non-governmental cultural organisations. Although all initiatives involve partnerships between cultural players and institutions from other sectors like health and education, no formal cross-sector institutional collaboration framework is in place. Also, there is yet little awareness regarding the impact of art on well-being (Bidwell 2014). Moreover, although culture is regarded as a catalyst for urban transformation processes (Cluj-Napoca Municipality 2014), the level of confidence in the potential of culture to propose effective solutions in the area of health and well-being, as well as the level of support for the cultural sector in general, are relatively low.

In this particular context, with a mandate to implement the culture-led development plan jointly created by various city stakeholders during the preparation of the bid for the European Capital of Culture 2021 (Cluj-Napoca European Capital of Culture Association (2016), the Cluj Cultural Centre, a non-governmental organisation for culture and sustainable development, has initiated the Inner Space programme, an interdisciplinary project exploring the relation between culture and well-being. The long-term view is to contribute through culture to an increased quality of life and well-being. The initiative also aims that Cluj-Napoca becomes one of the first cities from the Central and Eastern European region to include cultural well-being among the strategic factors for its sustainable development. To this end, the programme includes experimentation (pilot projects), research, advocacy and capacity building activities, informing urban and national policies.

## Case description

Burnout is a psychological condition which affects people in multiple ways (Salvagioni et al. 2017). Several studies confirm that burnout and depression are interdependent, each one having implications for the other (Maslach and Leiter 2016). The pandemic period was associated with high demands for individuals, which often led to reduced energy levels that affected psychological well-being and increased burnout rates (Nezich 2020; Meyer et al. 2021). The stress related to COVID-19 has been associated with poor mental health and a low quality of life, including burnout (Yıldırım et al. 2021). Working from home consisted in addressing family needs, taking care of children, and dealing with fears regarding their own health and the health of others, reduced social support, loneliness and financial insecurity (Yıldırım et al. 2021). The working environment is constantly changing and factors such as high workload, low salaries, workplace stress can increase the level of burnout (Irandoost et al. 2021). After the pandemic, remote working became the new approach of employees and new challenges are appearing in the working environment (Reynolds 2020; Ervasti et al. 2022).

Culture-based interventions can improve the cultural knowledge and sense of identity of the participants, and help them develop a positive attitude and change their behaviour (New Zealand Government 2017). These interventions can influence physiological, biological, and emotional health by stimulating emotions and behaviour change. They have the potential to enrich and enhance the memory, stimulate and accelerate learning, boost self-confidence, and decrease the level of pain. The individual can become more creative and the cohesion can increase between employees resulting in a more balanced/productive psychosocial work environment (Theorell et al. 2013).

In this section of the paper the phases of a pilot cultural intervention for burnout in Cluj-Napoca are presented, including a process evaluation. To prove the effectiveness of the pilot project, in the section are also presented the results of the qualitative and quantitative measurements of the impact of this cultural intervention on participants, by using several specific indicators.

### A pilot cultural intervention for burnout in the city of Cluj-Napoca

**Fig. 1:**
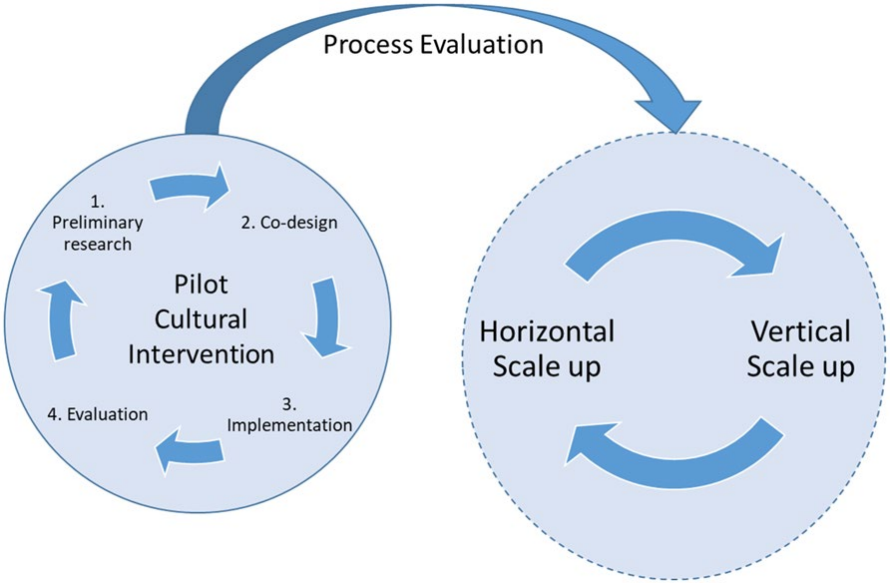
Cultural Intervention for Burnout: from pilot project to scaling up. Source: Authors' elaboration

With the view to understand the requirements and criteria for the development of a culture-based intervention in Cluj-Napoca, the Cluj Cultural Centre conducted a pilot project to design, implement, and assess a cultural intervention scheme addressing the growing health challenge of burnout. The pilot action was carried out within Art & Well-being (part of Inner Space programme), a project co-financed by the Creative Europe Programme of the European Union. The pilot implemented in 2020 provided a programme of creative workshops—including different types of art such as theatre, visual arts, music, writing (see Stuckey and Nobel 2010)—to eleven participants with burnout symptoms. The purpose of the activities was to help participants overcome burnout and increase their abilities to manage future symptoms (see Viding et al. 2015). They also sought to enhance imagination and emotional intelligence, reduce anxiety, improve self-esteem, and encourage self-expression. The workshops were planned and co-designed by practitioners and researchers in the fields of art and health and were facilitated by an artist with experience in cultural mediation. The process of the pilot cultural intervention implied four main stages: preliminary research, co-design, implementation and evaluation, as showed in (Fig. 1).

#### Phase 1. Preliminary research

This phase involved desk research with the purpose to gather relevant knowledge from existing studies on well-being effects of art interventions. The work was carried out by a team of health researchers from the Babeș-Bolyai University in Cluj-Napoca. More than 40 research articles, the majority from the field of public health and psychology, were reviewed. The research team developed a *search strategy* in order to retrieve accurate results, a set of *eligibility criteria for inclusion of publications* in accordance with the objectives of the project, and an *analysis grid* for organising and synthesising the findings. The findings supported the project team in establishing the intervention approach. The lead artist and the cultural experts brought an important contribution based on practical experience to this phase.

#### Phase 2. Co-design

The co-design phase aimed at creating a clear and precise blueprint for the actual intervention, detailing specific intervention objectives, cultural approaches, and a session-by-session work plan. The phase consisted in several group working sessions, involving researchers (one research assistant and three students from Babeș-Bolyai University), the lead artist and two cultural experts from the Cluj Cultural Centre.

The outcome of the co-design phase was an *intervention plan* combining cultural practice with findings from the preliminary research, providing guidance for the lead artist and the implementation team. The enrollment strategy and the instruments to measure the effects of the interventions were also elaborated.

The *enrollment strategy* detailed eligibility criteria for participation, selection criteria, the procedure, and documents of the call for participation. The call text informed participants on the research dimension of the project.

The *pre-intervention questionnaire* was designed to evaluate the most relevant aspects related to burnout and overall well-being. It included the following subsections: socio-demographics about participants; lifestyle habits such as smoking status, dieting, physical activity, medical diseases; cultural participation habits; general state of well-being measured through The Psychological General Well-Being Index, short version (PGWBI-S) (Grossi et al. 2006, 2012), The Connor-Davidson Resilience Scale (2-item version) (CD-RISC2) (Vaishnavi et al. 2007), The Scale of Positive and Negative Experience (SPANE) (Diener et al. 2009); and burnout measured through The Burnout Inventory (Maslach and Jackson 1981). Questions regarding the participants' expectations and motivation in relation to the programme and previous experience with arts/creative activities were included in the pre-intervention questionnaire.

The *post-intervention questionnaire* was adapted to include the following subsections: general state of well-being, burnout, resilience, their positive and negative experiences, if the participants’ expectations were met, the opinion regarding the experience and intentions of future engagement in similar activities.

The *follow-up questionnaire* (administered 18 months after the end of the intervention) aimed to assess the level of burnout, general well-being, resilience, positive and negative experiences, and cultural participation habits. It also included additional open-ended questions on: perceived changes in the participants’ way of relating to stress and negative emotions, coping strategies used over the past 18 months and perceived effectiveness of these strategies, changes in the way of relating to the arts and other effects (if any) of the programme in the participants’ lives. The participants were also asked to suggest recommendations for the improvement of the programme in the long-run and to mention to whom (if at all) they would recommend participation in the programme. Finally, they were asked if they would participate in a similar programme in the following scenarios: their employer recommends the participation and covers the costs, their physician/therapist recommends participation or they pay themselves the costs for participation.

In addition to quantitative measures, the evaluation methodology designed in this phase also included two focus group guides: (1) a focus group to better understand the experiences of the participants and the way they perceived and evaluated the cultural intervention, which included the following sub-categories: (a) questions regarding their expectations, (b) questions regarding the sessions and their well-being and (c) questions assessing the way in which the programme was organised and delivered; and (2) a focus group with the intervention team (lead artist, one researcher and two representatives of the Cluj Cultural Centre) to understand their perception on the effects of the intervention and to identify the strengths and the weaknesses of the project.

#### Phase 3. Implementation

The *enrollment* of participants took place online, with an open call launched on 15 September 2020. Information published in the call included: the aim of the intervention, how the intervention was going to take place, the workshops schedule, the maximum number of participants, eligibility criteria, selection criteria, and other details regarding the implementation and evaluation of the intervention. Permission to use their data for research and publication was included in the registration documents. The call for participation was distributed through the Cluj Cultural Centre’s communication channels (website, social media, newsletter) and through mass media (newspapers, radio shows, etc.).

*Registration* was open for 15 days. The results of the *selection* were published 3 days after the registration deadline. The selection was carried out by the core team involved in the co-design phase. The participants were selected based on the following *eligibility criteri*a: were aged over 18, confirmed their availability for all the seven sessions of the workshop, agreed to be part of the accompanying research, and reported high rates of burnout self-evaluated through Maslach Burnout Inventory (Maslach and Jackson 1981). Additionally, the selection took into account diversity and gender balance within the group, thus participants with diverse professional backgrounds and different ages were selected from the pool of eligible candidates. Twelve participants were invited to join the programme. Although during registration they have agreed with the conditions, one of the participants dropped out after the first session invoking lack of time to attend all the sessions. All the other eleven selected participants completed the programme.

The *cultural intervention* took place on seven consecutive Mondays in October and November 2020, within the premises of Cluj Hub, a centrally located co-working space. The activities took place in a large multifunctional room, which allowed flexible settings and compliance with COVID-19 social distancing rules. The intervention plan served as basis for implementation, and included the objectives, the number of sessions, the date for each session, the workshop activities, the expected outcomes and the COVID-19 safety protocol. The objectives of the creative workshops were focused on: O1. Awareness and assessment of burnout states (session 1, 2, 3); O2. Managing burnout states (session 4, 5, 6) and O3. Evaluation and prevention of burnout (session 7). The intervention included a wide range of art and creative activities, such as drawing, painting, music, theatrical improvisation, and photography. The creative activities proposed were adapted to the COVID-19 specific restrictions, avoiding direct contact between the participants and the use of the same tools, requiring a lot of spontaneity from the lead artist and the participants.

The selected participants were asked to complete a pre, post-intervention and follow-up questionnaire in order to measure the burnout and well-being rates. The *pre-intervention questionnaire* was filled in during the first session of the programme, when participants also signed an informed consent for voluntary participation in the research. After the last session, participants filled in *the post-intervention questionnaire* online and participated in a *face to face focus group*. The *focus group* with the project team was carried out online (on Zoom). The *follow-up questionnaire* was completed 18 months after the programme finished.

#### Phase 4. Evaluation

*Analysis of data collected through questionnaires* was performed by using IBM SPSS Statistics 25.0. A repeated measures ANOVA was performed for comparing the means of the variables of interest across the three temporal milestones (pre-intervention, post-intervention and follow-up). To *analyse the data from the focus groups*, the interviews were transcribed and a codebook was developed after reading the results. An inductive content analysis was performed by identifying different themes, categories and codes to establish patterns in our data.

The participants' sample consisted of a total of eleven participants, eight women and three men. They were professionally active in the fields of health, arts, IT, business management, education, marketing, and two were university students. Most of the participants were non-smokers (n = 8) and were not on a diet (n = 8); most of them were engaging in physical activity (n = 8), doing exercise 2–3 times per month (n = 1) or 2–3 times per week (n = 7). Half of them reported to have been diagnosed with a medical condition (n = 5). Among participants, one was a *professional artist,* while the others either had *some experience with art* or had *appreciation for art* prior to participating in the programme.

The findings based on the data collected *prior to the intervention* showed that some participants were experiencing *different types of negative emotions such as stress, depression, anxiety, numbness, sadness, and irritability*. These emotions affected their relationships with their colleagues and had a negative impact on their productivity. One participant also mentioned physical symptoms because of the intense emotions, reaching somatized stress, a common phenomenon for burnout. Some of the most encountered dysfunctional thoughts in the sample of participants were *perfectionism, high standards and a limited, narrow vision of the world*. These thoughts led to *negative behaviours such as procrastination*. Two participants mentioned they were *behaving impulsively and their behaviour surprised others* and them as well.

The *focus group findings (post-intervention)* showed that the group dynamics and the genuine interactions seemed to energise the participants and to lower their stress levels. The lead artist noticed changes in the state and behaviour of some participants: one participant managed to use her voice more powerfully by the end of the intervention, and another was able to accept her body and act confidently. After the intervention, participants chose to engage in more useful behaviours, compared to their previous dysfunctional behaviours. There were changes observed in their mindset, in the way they perceived and approached the world in general and burnout issues in particular. Participants enjoyed the variety of art techniques and exercises that involved performing arts, drawing, photography, dancing, music and drama. At home or even at their workplaces the participants practised photography or music listening as a part of their daily routine along with drawing which was assigned to them as homework. The results emphasised an increased interest in the world of art. The participants stated that if they were given the chance, they would participate in similar projects.

The *follow-up measures* (18 months after the end of the intervention) revealed that the participants perceive that burnout or stressful periods now disappear faster, that they better manage their states of tiredness and their reactions, identify the burnout states earlier, prioritise important aspects of life, such as family and use art as a coping strategy. As coping activities, they use art-acting, music playlists, drawing, painting, joinery-, gardening, physical exercise, psychotherapy and sleep. Some participants mentioned that they reduced social media use, are paying more attention to their body, and use dancing or music/sounds for relaxation/regulation. Some participants mentioned an increased interest in art making and participation. Most of them would participate in similar interventions. Some described the project experience as revealing, very useful, creative and with benefits which offered energy and support for a long period of time. Participants would recommend such intervention to college students, people working in the medical field, teachers, life partners and friends.

Based on the *quantitative measures*, by the end of the intervention, the level of burnout for all the participants decreased from *high* (76–125) to *medium* (51–75) or *low* (35–50). Out of the total of eleven participants, nine reported medium level of burnout after the intervention and two individuals had low levels of burnout.

A repeated measures ANOVA with a Greenhouse–Geisser correction determined that the mean burnout scores differed significantly between time points (pre-intervention, post-intervention and follow-up) F(2, 20) = 29.277, P < 0.001. Post hoc analysis with a Bonferroni adjustment revealed that the burnout score has significantly decreased from pre-intervention to post-intervention (30.46 (95% CI 17.5 to 43.3), decreased from pre-intervention to follow-up (21.0 (95% CI 11.36 to 30.63), but there were no significant differences between post-intervention and follow-up (−9.45 (95% CI −21.744 to 2.83), meaning that post intervention and follow-up scores were similar.

The same analysis determined that the general well-being (PGWBI) scores differed significantly between time points (pre-intervention, post-intervention and follow-up) F(2, 20) = 31.936, P < 0.001. The post hoc analysis showed that the well-being scores significantly increased from pre-intervention to post intervention (−9.00 (95% CI −13.98 to −4.01), increased from pre-intervention to follow-up (−10.09 (95% CI −13.14 to −7.03) but there were no significant differences between post-intervention and follow-up (−1.09 (95% CI −4.73 to 2.54), the scores being high in both assessments compared with pre-intervention.

Regarding positive experiences (SPANE-P) the scores differed significantly between the three assessments as well (2, 20) = 17.09, P < 0.001. Participants reported significantly higher scores from pre-intervention to post-intervention (−6.81 (95% CI −9.55 to −4.08) and higher scores between pre-intervention to follow-up (−5.00 (95% CI −8.79 to −1.20). There were no statistically significant differences between the positive experience scores from post-intervention to follow-up, both the time periods having a similar mean (1.81 (95% CI −1.95 to 5.58).

The affect balance (SPANE-B) differs significantly between the time points as well F (2, 20) = 10.763, P < 0.001. Participants reported significantly higher affect balance score between pre-intervention to follow-up (−9.09 (95% CI −16.66 to −3.15), compared to pre-intervention to post-intervention (−4.45 (95% CI −9.37 to 0.46) or from post-intervention to follow-up (−5.45 (95% CI −12.03 to 1.12).

The repeated measures ANOVA determined that the negative experiences differed significantly between time points F (2, 20) = 10.844, P < 0.001. The post hoc analysis revealed no differences in negative experiences from pre-intervention to post-intervention (−2.36 (95% CI −6.71 to 2.05), while between pre-intervention and follow-up (4.09 (95% CI 1.24 to 8.56), the negative experiences significantly decreased. Between post-intervention and follow-up, the negative experiences decreased as well (7.23 (95% CI 1.81 to 12.73).

**Fig. 2:**
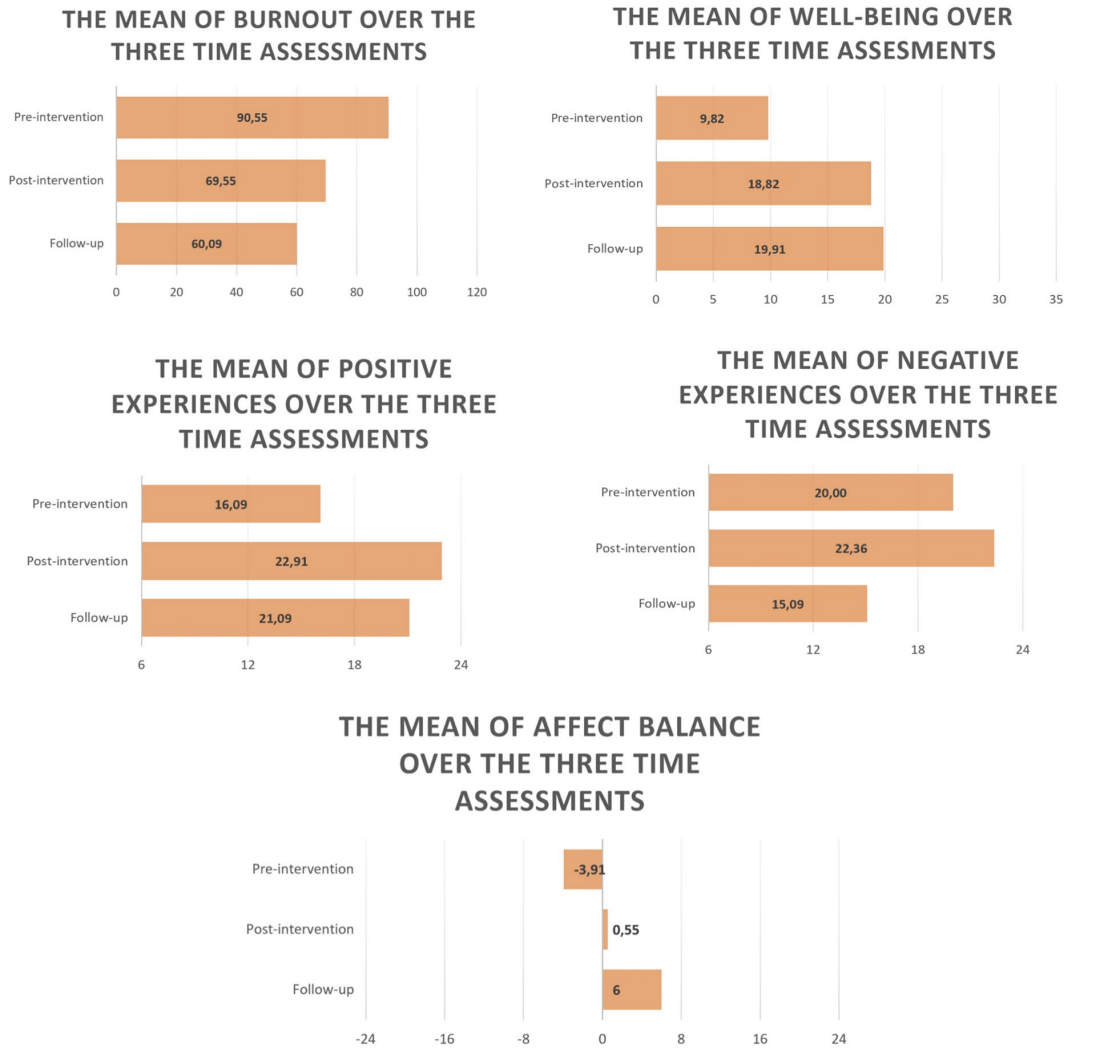
The evolution of well-being indicators over time (three time assessments) Source: Authors’ elaboration

The Fig. 2 shows the mean (M) of the well-being variables that were statistically significant after performing repeated measures ANOVA.

### Process evaluation of the pilot cultural intervention for burnout

To better understand how cultural interventions for burnout can be improved, the results of the evaluation of the overall process are presented in the following section.

Considering the motivations to enrol in the programme, seven different reasons were listed by participants: *description of the event, programme and the description of the activities, the fact that the activities were going to take place in a group setting, knowing the organisers and their previous activities, curiosity of the unknown, and the research component.* Familiarity with art seems to be an important factor, since five participants mentioned that having engaged before in similar artistic activities contributed to their decision to enrol. Some of the participants mentioned that the pandemic had an influence on their decision to join. However, most of them mentioned that they thought other people would be prioritised for selection for having a higher level of burnout. This may indicate that people do not necessarily have an accurate perception of their own degree of burnout, and also that anxiety to be rejected may need to be a factor to be considered in followup interventions.

In terms of expectations and if they were met or not, six participants said they *did not have any clear expectations before enrolling,* while two participants had *clear expectations,* based on the fact that they knew the artist and the host organisation and trusted the process guided by them. At the end of the programme, all the participants stated that their expectations were met or exceeded. They enjoyed the dynamic of the group, the creative activities and everything was better than they initially imagined.

The call for enrolment was distributed through different online channels and also relied on viral distribution of the announcement. The reputation of the organiser and of the artist influenced the decision to participate, as the participants already trusted them. The event description and the title seem to have been decisive for two of the participants' enrollment. A diverse positioning strategy for similar creative workshops provide opportunities for people with different motivations to attend. Future interventions would need to consider diversity of outreach channels and also possible different formats and language for the communication, i.e. use videos for younger audiences. Making reference to the reputation of the artists involved and to the experience and professional reputation of the organisers may also support interested individuals to enrol. Social media promotion is particularly suitable for people who are unfamiliar with this kind of experience.

In what concerns *the number of sessions and their frequency*, and the *day and time of the session*, a series of 7–10 weekly sessions, taking place in the afternoon, in the first part of the week seems a promising option for future iterations. The group size needs to be carefully decided, a group of 8–12 participants (Table 1) allows the lead artist to connect with each individual and to adapt the workshops to the specific needs identified for each participant. The lead artist was successful in the implementation of the intervention plan, although in some cases she has adapted some of the exercises to the group needs. Given the fact that the intervention took place during the COVID-19 pandemic, we also explored the participant’ intentions to participate in similar projects online and most of them responded that they prefer the physical, face to face meetings.

**Table 1:** Well-being Outcomes—Synthesis of qualitative assessment Source: Authors' elaboration

Emotional states described by participants		Quotes
Before the intervention	Depression	‘I felt a lot of sadness, I didn't want to interact with anyone anymore, I practically couldn't find the small joys of life and I felt that it’s not good, that I’m not well.’ (Female, 36–45 years old, IT specialist)
	Dissatisfaction	‘I think that burnout for me meant a kind of continuous dissatisfaction and contempt.’ (Female, 19–24 years old, student)
	Anxiety	‘…thoughts, I mean I was blocked in my mind, I was staying in my mind for a very long time.’ (Female, 25–30 years old, Team Lead)
		‘I felt very anxious because I often feel quite uncomfortable in unfamiliar situations, and I don't know how to react and I end up reacting funny.’ (Female, 19–24 years old, Freelancer Digital Marketing)
	Somatization	‘Anxiety, stress, actually physical, which manifests itself physically.’ (Female, 25–30 years old, Team Lead)
	Anger, impulsivity	‘…quite a few resignations from impulsivity, dismissals also from impulsivity, raising the tone at people without realising that I am doing this, a very accentuated state of nervousness.’ (Female, 31–35 years old, Project Manager)
After the intervention	Safety	‘I realised that no one cares and that everyone cares about their own thoughts, and they were thinking how they experienced in general the exercises and methods, they will not wait to see what no. 3 does.’ (Female, 25–30 years old, Team Lead)
	Acceptance	‘…and in relationship with my colleagues there were always pleasant emotions, I felt that I was accepted, that I was appreciated, I did not experience pressure or shame for something I did, they were really positive emotions.’ (Female, 36–45 years old, IT specialist)
		‘And regardless of the emotion, whether it is positive or not, I try to be present in that state and just let it be.’ (Female, 19–24 years old, Freelancer Digital Marketing)
	Motivation	‘I think what has changed now is what I do when I feel "overwhelmed", I mean, when I feel the burnout, because sometimes I feel it, sometimes I just live with it, but when I feel it, now I try to do a certain thing maybe to improve my state or to pass it. […] to do something, I don't know, to get up from the office, to go get another coffee or tea, to go and caress the cat, "I don't know, anything", something to get me out of that thing.’ (Female, 25–30 years old, Team Lead)
Follow-up	Coping strategies	‘I’m trying to apply the idea that the only control we have is over our reactions and actions, and less over the situation. When I’m feeling overwhelmed I try to reach out to other people for help, even if it still feels like a burden to them.’ (Female, 25–30 years old, Team Lead)
		‘I learned to prioritise things better and to dedicate my energy to the things that are important to me.’ (Male, 25–30 years old, IT specialist)
	Stress management strategies learned	‘The stress decreases and I no longer reach the end of my strength.’ (Female, 19–24 years old, Freelancer Digital Marketing)
	Art-based coping activities	‘I've been trying to consume better quality media instead of endless scrolling, recreational activities are now more about taking a day off and listening to music/dancing.’ (Female, 25–30 years old, Team Lead)
		‘Art is the antidote to fatigue.’ (Female, 31–35 years old, Project Manager)
	Lasting effects of the intervention	‘Some effects are felt, but the energy of the group is missing.’ (Female, 36–45 years old, IT specialist)
		‘So far the benefit is the awareness that everything is under control.’ (Female, 31–35 years old, Project Manager)

Participants found the exercises focused on *relaxation, emotional awareness,* and *mindfulness* particularly useful. A few of attention, relaxation and creativity exercises were recommended as part of the daily routine of the participants. One practice derived from the workshop series that both the lead artist and the participants found of value, was to create music playlists resonating with different emotional states, which they can later access at will. This, along with other practices, came to reinforce and support long-term adoption of some of the useful behaviour/rituals developed within the programme. The artist expressed confidence that *this type of art-based approach would benefit people with other emotional issues* (e.g. depression, anxiety) too, while highlighting that the implementation plan would need to be adapted, based on research, to the specificities of each condition. Overall, the participants mentioned they had a good relationship with the artist. In what concerns *the artist’s experience and interaction with the participants,* she felt challenged to find balance between participants, as some of them were more extroverted and energetic, while others needed more time to process the information and get involved in the exercises. She also felt some resistance from older participants when they had to perform individually on a certain subject, while everyone felt comfortable in group exercises.

*Working with people with burnout symptoms*, the artist noticed in the first sessions that she could not properly read the emotional states of some of the participants. She was not sure if they were anxious, introverted or scared, therefore she chose to act more cautiously in the beginning. The artist also mentioned that the groups she usually works with, mostly young people and adults who engage in creative workshops out of interest for art and creative self-expression, are more energetic than this group manifesting symptoms of burnout. On the other hand, she found this particular group was more reflective. The pandemic had an influence on participants, the artist noticing recurring thoughts and behavioural patterns related to the COVID-19.

Having in mind a possible scaling up and replication of this type of cultural intervention, the team aimed to identify the skills and the prerequisites for an artist or facilitator. According to this experience, the artist needs to have: a well-established art practice and/or robust knowledge of the art discipline(s) employed, cultural mediation or facilitation skills and experience, and good coordination skills in order to follow the implementation accurately. Ease and skill in handling human interactions and sensing group dynamics, a positive disposition, flexibility, curiosity, playfulness and leadership are also useful traits for a facilitator. The capacity to be continuously present and connected with the participants proved to be highly relevant in the practice.

The recommendation of the participants for the long-lasting effects of the intervention was to repeat the experience also in the non-pandemic context, maybe during the weekend or once a year.

Such interventions create a sense of belonging and the awareness necessary to change dysfunctional behaviours, participants stating at the end of the programme that they associated the Mondays with the creative workshops. Creating a safe place for exploration encourages creativity and relaxation, while receiving proper guidance supports participants in managing and understanding their own feelings and behaviours. Spontaneity and openness from the artist help create the desired dynamics within a heterogeneous group and open doors for yet unexplored territories for participants.

## Discussion and future directions

Beyond assessing the effects of the intervention on the physical and emotional states of the participants and the overall process of the cultural intervention, an evaluation of the pilot programme was necessary in order to identify how such interventions can be expanded. The next section presents lessons learned from the pilot project and considerations for scaling up.

### Takeaways from the pilot intervention for future initiatives of its kind

This paper aimed to distil the ingredients of this pilot project that constitute the base for replication and adaptation of such culture-based interventions. Based on this experience, we recommend that future iterations observe a similar format, including preliminary research, co-design, implementation and assessment.

Knowledge is the key for getting such an intervention started. One needs to begin by aiming to get a thorough understanding of the issue/condition to be addressed, of the needs behind it, but also of the specificities of the local context. Acting without prior knowledge acquired from specialised literature or from practical experience poses possible risks.

The co-design of the interventions may be time consuming, yet it plays an important role for an accurate cultural-intervention plan. The experience of contributors with various backgrounds provides important insights for a specific programme design. The contributions need to be collected and documented in a structured manner in order to be further valuable in practice. The output of the co-design phase is the intervention plan, defining the objectives for each session, identifying the art-based exercises to be performed, and the expected outcomes. The plan provides a framework for a structured approach during the implementation, but flexibility in implementation is recommended.

The pilot project proved that a programme customised to the needs of the target group is required in order to ensure benefits for participants, as also stated in another research by Morris and Paris (2021). In group settings, combining group activities with individual activities may be an important factor for achieving expected outcomes, as suggested also by Shenaar-Golan and Walter (2018). Aiming to deliver an experience that expands way beyond the intervention sessions by equipping participants with creative tools and exercises that they can later practice at home, at their workplace, in nature, or whenever they feel that the acquired behaviour is useful for their condition needs to be a core element of the intervention design. Culture-interventions oriented towards well-being can provide a particularly supportive context for developing resilience and resilience-related skills (Zarobe and Bungay 2017).

Organisers of future interventions are suggested to offer an alternative to those who register and are not selected, be it a single-session intervention, a toolkit of resources that individuals could use at home or referral towards other cultural programmes available in their community.

Considering the accompanying research, a method combining quantitative and qualitative measurements proved appropriate. A documentation of the entire process is highly recommended. Future programmes aiming to assess the effects of participation in such creative workshops on peoples’ states may derive better insights by including a control group with similar profiles as the enrolled participants in their research. Additional neuroscience research could be very valuable in measuring the impact of these kinds of interventions, but such research requires substantial financial resources.

The availability of resources is a key aspect to have in mind for further interventions in order to make all the phases of the process possible and to deliver adequate conditions for implementation such as spaces, supplies, supporting materials and equipment, communication and dissemination means, and equitable payment for the entire team. Recruiting team members with the appropriate human and professional skills is key to the programme success. Particular attention needs to be paid to the skills of the artist(s) and process facilitators involved in direct relation with participants.

Scaling up of such interventions which mobilise knowledge, resources and skills from multiple disciplines require a high level of cooperation among different individual and institutional actors. Productive collaboration frameworks may significantly reduce the costs of implementation—by ensuring certain conditions like spaces, materials and equipment through in—kind contributions—and are key to the sustainability of culture-based well-being and health interventions. In an ecosystem like Cluj-Napoca, with a strong collaborative culture, getting more institutions onboard for new iterations of the piloted intervention should be rather easy to accomplish. The challenge we foresee is to get financial and policy-level support for to scaling up this or similar culture-based interventions.

### Scaling up cultural interventions for well-being in Cluj-Napoca

The aim of the process evaluation was to identify the conditions for scaling up this particular intervention from pilot level to community level. This would imply a large-scale programme for people with burnout or at risk of burnout, using the blueprint developed and tested within the pilot project, that would be available continuously or at least regularly in the city of Cluj-Napoca. Considering the fact that this was a single and first activity of its kind in the city, there is a long way to go to achieve this level. Designing a scaling up strategy involves making strategic choices about the types of scaling up to be used (World Health Organization and ExpandNet 2010).

The concept of upscaling is elaborated in different types of literature: transition management, business studies and development studies (Van Winden 2016). In the present paper we operate with the concept of scaling up defined in the development studies (Van Winden 2016). The scaling up is defined as “expanding, adapting and sustaining successful policies, programmes or projects in different places and over time to reach a greater number of people” (in Hartmann and Linn 2008, p.8). Simmons et al. (2007) defined scale up for the health sector as “deliberate efforts to increase the impact of health service innovations successfully tested in pilot or experimental projects so as to benefit more people and to foster policy and programme development on a lasting basis” (in Hardee et al. 2012, p.2).

According to Hardee et al. (2012), in practice scaling up may take different formats, such as: spontaneous scale-up and planned scale-up models which imply **vertical scale-up**—institutionalising an innovation at the national or sub-national level through policy, political, regulatory, budgetary, or other health system changes; **horizontal scale-up** or “spread” or “quantitative scaling”—replicating an intervention in different geographic sites or expanding it to a wider area; and **functional scale-up** or “diversification”—testing or adding a new innovation to an existing one. On the other hand, Hartmann and Linn (2008) make a clear distinction of the three institutional approaches for scaling up development interventions: (1) hierarchical, which involves top-down, planned programmes driven by strong central leadership, such as immunisation and literacy campaigns; (2) individualistic, which implies a bottom-up action, in which effective development is the result of individuals’ actions; and (3) relational, which involves the accumulation of social capital through decentralisation, participatory methods and empowerment technique; and among three organisational paths: (a) expansion; (b) replication; and (c) spontaneous diffusion. Cooley and Kohl (2005) also propose the following types of scaling up: expansion, replication and spontaneous diffusion (in Winden 2016). According to them, expansion is scaling up a pilot within the organisation that developed it; replication is scaling up by another organisation than that originally developed the initial pilot; and spontaneous diffusion is the spread of good practices of their own (in Winden 2016). World Health Organization (2016) suggests that expansion means to extend the organisational structures and/or service provision (geographical expansion, expansion of population reached), while replication involves implementing new, innovative or good practices in other, more or less independent organisations and settings. In a scale-up process focused only on the expansion of an intervention (horisontal) or when interventions are not aligned with existing policies and systems, sustainability is compromised (Spicer et al. 2018; Wickremasinghe et al. 2018, in Bulthuis et al. 2020).

In scaling up health interventions, various theoretical models are used, in accordance with the challenges and complexity of the projects. Milat et al (2015) synthesised public health scaling up models and frameworks: (a) **Scaling up population health interventions, New South Wales Ministry of Health**—a 4 step process: (1) assess scalability assessment, (2) develop a scaling up plan, (3) prepare for scaling up, (4) scale up; (b) **9 steps to scaling up, WHO ExpandNet**: (1) planning actions to increase the scalability of the innovation; (2) increasing the capacity of the user organisation to implement; (3) assessing the environment and planning actions to increase the potential for success, (4) increasing the capacity of the resource team to support scaling up, (5) making strategic choices to support vertical scaling up (institutionalisation); (6) making strategic choices to support horizontal scaling up (expansion/replication); (7) determining the role of diversification, (8) planning actions to address spontaneous scaling up, and (9) finalising the scaling-up strategy and identifying next steps; (c) **Scaling up management (SUM) framework**—3 steps process: (1) developing a scaling up plan; (2) establishing the preconditions for scaling up and (3) implementing the scaling up process.

According to Milat et al. (2015) the success factors for scaling up public health interventions are: establishing monitoring and evaluation systems; costing and economic modelling of intervention; active engagement of implementers and the target community; tailoring scale-up approach to local context and use of participatory approaches; systematic use of evidence; infrastructure to support implementation such as training, delivery systems, technical resources; strong leadership; political will; well-defined scale-up strategy; strong advocacy; flexible responses to human resource constraints; formative research to ensure appropriate intervention design; equity of intervention delivery and monitoring consequences across socio-demographic profiles; effective communication strategy; effective governance and coordination; clear role definition; keeping the intervention model simple; financing models; programmes are visible and effectively packaged; developing strategies for integration into existing services. Additionally, Milat et al. (2015) list the following barriers to scaling up public health interventions: not adapting intervention approaches to the local context; intervention costs and other economic factors; lack of human resources; resistance to the introduction of new practices; insufficient investment in implementation (training, monitoring and evaluation systems); staff recruitment and staff turnover; lack of political will; traditional research funding processes are not flexible enough to support evaluation of scale up; leadership changes amongst implementation agencies; poor engagement with stakeholders; poor role delineation and maintaining quality and consistency of health interventions at scale.

Cooley and Kohl (2005) recommend that scaling up should only take place after an effective and efficient evaluation of the model/pilot conducted on a limited scale. Also, they recommend adapting and, where appropriate, simplifying the model in order to focus on those aspects critical to its successful scaling up.

For this initiative the process of scaling up has not started yet, but very brief directions for this are identified in this paper. The pilot testing was successfully completed, effects being measured both at the end of the intervention and 18 months later. Regarding the participation in such a program, 7 participants out of 11 who completed the follow-up mentioned that they would certainly participate if the employer would recommend the participation and cover the costs, 7 participants would certainly participate if the physician or therapist would recommend it and 4 participants would maybe participate if they would need to cover the costs by themselves along with 4 participants who reported that they would very probably participate in such a case.

Considering possible scale-up pathways, the Cluj Cultural Centre envisages a replication of this pilot project in another organisation. In this respect, the cultural interventions can be delivered by cultural players within healthcare institutions, schools, community centres or companies, the costs being covered by the host partners. Even if the sample of the pilot cultural intervention participants is not representative of the entire active population of Cluj-Napoca, the focus of the project becomes increasingly relevant for stakeholders, such as business companies and universities. Given the particular business ecosystem of Cluj-Napoca, large companies, especially multinational companies, are more likely to use the programme first for their employees and cover the costs from their human resource budgets. Burnout and mental health conditions are growing in the current context and institutions increasingly focus on possible strategies to overcome these public health issues (Jagodics and Szabó, 2022; Menon et al. 2022). This scenario seems more feasible to happen in the city as there is a perceived need. Multinational companies seem eager to respond to workplace challenges through innovative strategies. They also have the implementation capacity, with human resources departments taking charge of the well-being of the employees and corresponding budget allocations. The tools to measure burnout and effectiveness of art-based interventions developed in the pilot initiative can be used by companies to measure the impact on their own employees. For the programme to be operational in a company it is important to have an internal coordinator to mediate the relationship between employees and cultural players. In terms of norms, practices and resources, companies should develop long-term employee well-being strategies and dedicate budgets to culture-based activities within these strategies. Focus of strategies should change from offering employees opportunities for entertainment to providing them with educational, health and well-being benefits (Gritzka et al. 2020). Culture-based well-being activities do not require complex logistics, a spacious room, basic supplies and refreshments would in most cases suffice. This type of programme can provide, besides increased well-being, a deeper understanding and engagement with the arts from both companies and employees. Additionally, they can facilitate an improved work environment/atmosphere by increasing team bonding and a sense of belonging. Possible constraints may include reluctance to participate from certain employees as well as preference towards other type of activities. These may be overcome by offering activity previews or trial periods. Following the guidelines provided by theoretical models, a more detailed scaling up strategy will be defined in the future.

On a long-term run, a functional mechanism for culture/arts on prescription (see Jensen 2019; Holt 2020) in the city of Cluj-Napoca is suggested and it should provide (1) delivery of cultural intervention programmes for health and well-being, (2) referral pathways for people in need, (3) support systems, including funding, for the entire value chain (Cluj Cultural Centre 2021). These conditions can be set into motion through different scenarios. For instance, the local administration provides funding to cultural organisations to deliver the interventions in partnership with health institutions which recruit and refer participants. In a different scenario companies contribute to the establishment of a local fund that would support an art on prescription mechanism, ideally through a joint fund with local/central authorities. A more sustainable approach would be the integration of such services in the welfare system, allowing for cultural activities to be prescribed by physicians, therapists, school counsellors, HR departments or social assistants. This type of policy requires mobilising political will and support at national level.

## Conclusions

The use of art as a method for improving well-being, managing emotions, enhancing the freedom of expressing ideas and values is becoming more significant in the context of prevalence of mental health disorders (Jensen and Bonde 2018; Fancourt and Finn 2019; Sacco et al. 2020; Karkou et al. 2022). Experimental cultural initiatives aimed at improving health and well-being prove their worth and represent essential opportunities for innovation in contexts of urban development. Their careful documentation and research may provide very useful insights into new, effective, and accessible ways to improve quality of life and to help manage the unwanted effects of rapid urban development and of global challenges (such as the climate crisis and the pandemic) on people's physical and mental health, but they only represent a very first step. As long as efforts and support are limited to experimental action, without commitment for the full development of interventions into long term integrated programmes and policies, the desirable outcomes at a macro level cannot be achieved. Funders should be aware of the potential of arts to contribute to individual and community well-being and provide adequate funding programmes that could be accessed by different organisations. They should also be aware that the real effects of such cultural interventions will be visible in the long run, thus expecting immediate results may prove deceptive. Culture-based interventions are often less expensive than other health and well-being support measures, thus contributing to increasing the quality of life in cities, and possibly leading to budget savings on the long run.

The cultural intervention pilot implemented by Cluj Cultural Centre proved effective in relation to the proposed objectives of reducing levels of burnout, increasing levels of well-being, supporting participants to manage burnout associated symptoms and acquire constructive behaviours, the effects being visible and present even 18 months after its ending. The evaluation of the cultural intervention effects on participants' level of burnout showed promising results. All people all people showed decreased levels of burnout from pre-intervention to post-intervention, with a slight increase in the burnout level at follow-up, which may be attributed to many external and internal factors. Interest in engaging with the arts has grown in participants.

The evaluation of the overall process also showed satisfactory results. Research, co-design, working in an interdisciplinary team and using scientific evidence combined with practical experience to inform the project design were aspects of significance in this initiative. The intervention plan was appropriately designed and its implementation observed the blueprint to a great extent, minor adaptations were performed in order to accommodate the level of energy and dynamic of the group. Art-based exercises and guided group interaction were the key design elements of this intervention and they have proven both effective and efficient. Given the growing number of individuals that suffer from burnout or are at risk to develop burnout, such interventions have a high potential to produce a positive impact. Considering that the cost of the pilot included an intensive co-design and research effort, future iterations would be possible at a significantly lower cost. While this pilot programme was oriented towards treating existing burnout symptoms, future programmes need to also address burnout prevention. To this view, the intervention plan needs to be carefully re-designed and adapted.

Based on the results, the most suitable format to systematically deliver such interventions in a city like Cluj-Napoca is to establish a local administrative collaboration protocol (see Laitinen et al. 2020) and the support framework for a local programme of arts on prescriptions on the long run. The collaborating structures need to include stakeholders from culture, business, and health sectors, local authorities at city and metropolitan level, universities as well as companies and other civil society actors. For the time being, there are systemic gaps that need to be addressed. Measures are needed to raise awareness on the potential of culture to support individual and community well-being, draw attention to the added value and the effectiveness of culture-based health and well-being interventions, create opportunities for capacity development for artists, cultural players and other professionals involved in such interventions, activate local partnerships and mobilise financial support.

## Data Availability

The datasets used and/or analysed during the current study are available from the corresponding author on reasonable request.
